# Geographic origin and wheat variety shape microbial and functional profiles of brewing wheat for Daqu fermentation

**DOI:** 10.3389/fmicb.2026.1879363

**Published:** 2026-07-24

**Authors:** Hao Tang, Shouhang Zheng, Yao Lai, Qin Wang, Ning Yang, Na Luo, Li Cai, Ying Liu, Xinqi Chen, Yaqi Yang, Hongshen Wan, Zehou Liu, Yang Li, Fan Yang, Wuyun Yang, Yong Ren, Jun Li

**Affiliations:** 1 Crop Research Institute, Sichuan Academy of Agricultural Sciences, Chengdu, China; 2 Crop Germplasm Innovation and Genetic Improvement Key Laboratory of Sichuan Province, Chengdu, China; 3 Triticeae Research Institute, Mianyang Academy of Agricultural Sciences, Mianyang, China; 4 Nanchong Seed Management Station, Nanchong, China; 5 Key Laboratory of Wheat Biology and Genetic Improvement on Southwestern China, Ministry of Agriculture and Rural Affairs, Chengdu, China; 6 Biotechnology and Nuclear Technology Research Institute, Sichuan Academy of Agricultural Sciences, Chengdu, China

**Keywords:** wheat, Daqu, microbial diversity, grain quality, Daqu fermentation

## Abstract

**Introduction:**

Wheat is the primary raw material for traditional Baijiu Daqu fermentation, yet its role as a carrier of functional microbiota and its contribution to Daqu quality remain poorly understood.

**Methods:**

A total of 135 wheat samples representing five geographic regions and nine cultivars were subjected to sensory evaluation, physicochemical analysis, and 16S rRNA gene and ITS amplicon sequencing. Microbial community composition, predicted functional potential, and their associations with wheat quality traits were analyzed using PICRUSt2, Mantel tests, and correlation analyses.

**Results:**

Sensory evaluation indicated that the cultivation environment had a greater impact on Daqu quality than wheat cultivar, with wheat from the Dayi region and the MM916 cultivar exhibiting the most favorable characteristics. Sequencing identified 1,732 bacterial and 484 fungal amplicon sequence variants (ASVs), revealing significant geographic and varietal differences in microbial communities, while core taxa dominated by Pseudomonadota and Basidiomycota were consistently detected across all samples. Functional prediction suggested that microbial communities were primarily enriched in metabolic pathways, particularly carbohydrate metabolism, energy metabolism, and cofactor and vitamin metabolism. Spatial variation was observed in starch and sucrose metabolism, acetoin biosynthesis, and enzymes such as *β*-glucosidase and alcohol dehydrogenase. Wheat quality traits, especially protein, starch, and wet gluten content, were significantly associated with microbial composition and predicted functions. Thirty-two genera, including *Sphingomonas*, *Pedobacter*, and Martelella, showed strong correlations with these quality traits.

**Discussion:**

Geographic origin and wheat cultivar jointly shape the microbial communities and functional potential of brewing wheat, providing pre-existing microbial resources that may influence early Daqu fermentation and flavor formation. These findings offer a microbiome-based framework for evaluating and selecting high-quality wheat for Baijiu production.

## 1 Introduction

Baijiu production is an important traditional fermentation industry in China, and Sichuan Province is one of its principal production regions, with an annual production volume reaching 3.64 × 10^9^ L in 2021. The concentration of Baijiu-producing enterprises in Sichuan creates a substantial and stable demand for wheat used in Daqu preparation. Daqu production represents a complex “grain–microbe–enzyme” system in which microorganisms originating from raw materials and the production environment contribute hydrolytic enzymes, flavor precursors, and aroma compounds during fermentation (Jin et al., 2017; Wang et al., 2021). Therefore, understanding the sources and assembly of the Daqu microbiota is important for improving fermentation stability and Baijiu quality.

Culture-independent 16S rRNA and internal transcribed spacer (ITS) amplicon sequencing has substantially improved our understanding of microbial succession during Daqu fermentation (Han et al., 2024; Zhang et al., 2024). Previous studies have shown that mesophilic bacteria, including *Pantoea* and *Cronobacter*, tend to colonize during the early fermentation stage, whereas thermotolerant taxa such as Bacillus gradually become dominant as the temperature increases (Kang et al., 2022; Quan et al., 2023). *Bacillus* and other functional microorganisms contribute to saccharification, liquefaction, and flavor formation through the production of hydrolytic enzymes and volatile metabolites such as organic acids and pyrazines (Zou et al., 2018; Ren et al., 2023). However, most studies have focused on microbial succession after Daqu fermentation has begun, while the initial microbial inocula introduced by wheat raw materials have received considerably less attention. As the principal substrate and microbial carrier in Daqu production, wheat harbors diverse epiphytic and endophytic microorganisms that may influence the initial assembly and subsequent succession of the Daqu microbiota.

The large and concentrated Baijiu industry in Sichuan generates a substantial demand for wheat used in Daqu production. However, local wheat production alone cannot fully satisfy this demand, and the procurement of wheat from neighboring provinces and other wheat-producing regions has consequently become an important component of the raw-material supply chain. Thus, although the practical demand for high-quality Daqu-making wheat arises primarily from the Sichuan Baijiu industry, the geographic scope of wheat raw materials is not restricted to Sichuan. Wheat supplied from different regions is exposed to distinct climatic conditions, soil environments, cultivation practices, and disease pressures, all of which may shape its physicochemical properties and associated microbial communities (Deng et al., 2025; Zhou et al., 2025). These regionally differentiated microorganisms constitute potential initial inocula for Daqu fermentation and may influence subsequent community assembly through ecological competition and metabolic interactions. Therefore, understanding the geographic variation in wheat-associated microbiota is essential for evaluating raw materials from cross-regional supply sources and for improving the stability and quality of Daqu production in Sichuan.

Although the physicochemical properties of brewing wheat, including starch characteristics and protein structure, have been extensively investigated (Banu and Aprodu, 2015; Alfeo et al., 2021), the effects of wheat origin and genotype on indigenous microbial communities and their potential relationships with Daqu quality remain poorly understood. In this study, nine wheat varieties relevant to Sichuan Baijiu production, including local cultivars, brewing-specific lines, and disease-resistant germplasm, were cultivated in five representative ecological regions. Wheat-associated bacterial and fungal communities were characterized using 16S rRNA and ITS amplicon sequencing. We aimed to determine the relative effects of geographic environment and wheat variety on microbial community composition and to evaluate the relationships between wheat-associated microbiota and Daqu-making quality. These results provide a microbiological basis for the selection and cross-regional procurement of wheat raw materials and may facilitate more precise management of the initial microbial inputs used in Daqu production.

## 2 Materials and methods

### 2.1 Sample collection

Nine wheat varieties commonly used for Daqu production were selected, including historically utilized brewing varieties, local cultivars, newly developed brewing-specific lines, and high-quality disease-resistant varieties. These included Shumai 1671 (SM167), Chuanmai 618 (CM618), Mianmai 902 (MM902), Chuanmai 93 (CM93), Zhongkemai 1816 (ZKM1816), Mianmai 916 (MM916), Chuanmai 1694 (CM1694), Fanmai 8 (FM8), and Mianmai 907 (MM907). The varieties were cultivated in five representative regions: Xinyang, Henan (114.07°E, 31.63°N); Guiyang, Guizhou (106.71°E, 26.57°N); Hefei, Anhui (117.28°E, 31.86°N); Mianyang, Sichuan (103.75°E, 30.70°N); and Dayi, Sichuan (103.51°E, 30.58°N). After harvest in the following year, the wheat samples were transported to the food microbiology laboratory in sterile sampling bags at room temperature. Samples were immediately ground into powder, prepared in triplicate, and stored at −80 °C until further analysis.

### 2.2 Daqu preparation

Daqu was produced from September to December at Jinpeng Food Co., Ltd., Mianzhu, Sichuan Province, China, using the traditional *Baobaoqu* process. Fermentation temperature was allowed to vary naturally with the local ambient temperature without artificial temperature control, and environmental relative humidity was not monitored. All wheat materials were processed by the same experienced technician with more than 20 years of Daqu-making experience to minimize operator-related variation. Before milling, wheat grains were crushed using an HJA12-20 small double-roller mill (Qufu Hengjia Agricultural Machinery Co., Ltd., China), with the milling degree adjusted so that particles retained on a 20-mesh sieve accounted for more than 65% of the ground material. Water was then added and thoroughly mixed to obtain a final moisture content of approximately 40%. The mixed material was loaded into a rectangular mold with internal dimensions of 43 cm × 24 cm × 5 cm and compacted by foot into Daqu bricks. The center of each brick was shaped higher than its edges, with a maximum height of approximately 10.8–11.5 cm, while maintaining a smooth surface and compact internal structure. After demolding, the bricks were air-dried until the surfaces became slightly dry and were subsequently transferred to the fermentation room for arrangement. The bricks were covered with burlap sacks. During periods of excessively low winter temperatures, hot water at 60–80 °C was sprayed inside the fermentation room to increase the ambient temperature and humidity. The first turning operation was conducted approximately 24 h after the bricks were placed in the fermentation room, followed by one turning per day. After four consecutive daily turnings, water spraying was suspended for 1 day and the bricks were inverted and placed vertically. Water spraying was then suspended for an additional day during the subsequent fermentation period. Each brick underwent six turning operations in total. During each turning, bricks located near walls, doors, and windows were exchanged with those positioned in the interior of the room to reduce spatial variation in temperature and humidity. After completion of the turning stage, approximately 8–10 days after fermentation began, the bricks were stacked in at least two layers to promote drying and hardening. After approximately 30 days, when the bricks were completely dry, they were stacked in multiple layers and stored for 3 months before sensory evaluation. During microbial cultivation and fermentation, a wireless temperature sensor was inserted into the center of representative Daqu bricks, and the core temperature was recorded at 2-h intervals. The core temperature was maintained at 50 ± 7 °C, corresponding to the temperature range of medium- to high-temperature Daqu. Daqu bricks prepared from all wheat varieties were transferred into the fermentation room on the same day and subjected to identical fermentation, turning, drying, and storage conditions.

### 2.3 Sensory evaluation of Daqu quality

The sensory quality of Daqu prepared from different wheat samples was evaluated based on three attributes: appearance, cross-section, and aroma, with a total score of 60 points. Each attribute was assigned specific scoring criteria and evaluation standards. The appearance attribute accounted for 10 points, with full marks awarded to samples exhibiting a clean and intact surface without cracks or damage, a grayish-white to brownish color, and a crust thickness of less than 1.0 cm. The cross-section attribute accounted for 30 points, with full marks given to samples showing a uniform and smooth cross-section, dense and evenly distributed mycelia, an overall grayish-white color, and no defects such as black spots, ring-shaped dark zones, or fissures. The aroma attribute accounted for 20 points, with full marks assigned to samples presenting a typical strong-flavor Daqu aroma characterized by a mellow and pure fragrance, free of off-odors such as sourness or moldiness. Sensory evaluation was conducted in accordance with the Chinese industry standard QB/T 4257-2011 *General Analytical Methods for Brewing* Daqu, supplemented by enterprise-specific quality control criteria and traditional evaluation systems for high-quality Daqu. The evaluation of Daqu samples was conducted by one quality-control specialist from the Daqu-producing enterprise who had more than 20 years of professional experience in Daqu production and sensory assessment. For each wheat variety, five Daqu bricks were initially prepared. After fermentation, drying, and storage, three intact bricks were selected to avoid the influence of bricks accidentally damaged during turning or handling. The selected bricks were assigned random steel-stamped codes so that information regarding wheat variety and production origin was concealed from the evaluator during scoring. Each of the three coded bricks was evaluated separately for appearance, cross-sectional characteristics, and aroma. The mean score obtained from the three bricks was used as the final sensory score for the corresponding wheat variety, thereby providing three repeated measurements for each treatment.

### 2.4 Determination of physicochemical properties of Daqu

Physicochemical properties of Daqu samples, including moisture content, total acidity, liquefaction power, saccharification power, and fermentation power, were determined in triplicate according to the Chinese industry standard QB/T 4257-2011 *General Analytical Methods for Brewing* Daqu. The determination methods are described as follows: (1) Moisture content was determined by weighing approximately 4.5 g of sample into a pre-dried weighing dish, followed by drying at 105 °C for 4 h to constant weight. After cooling in a desiccator, the sample was reweighed and moisture content was calculated. (2) Total acidity was measured by weighing approximately 10 g (±0.001 g) of Daqu into a 250 mL beaker, adding 200 mL distilled water, and stirring magnetically at room temperature for 15 min. After standing for 15 min, the mixture was filtered through absorbent cotton. A 20.0 mL aliquot of filtrate was diluted with 30 mL distilled water and titrated with 0.1 mol/L NaOH using an automatic potentiometric titrator to an endpoint of pH 8.2. Distilled water was used as a blank, and total acidity was calculated based on NaOH consumption. (3) Liquefaction power was determined by weighing a sample equivalent to 10.00 g dry basis (±0.01 g), adding 20 mL buffer (pH 4.6) and 180 mL distilled water, and extracting in a 35 °C water bath for 1 h to obtain a 5% enzyme solution. A reaction mixture containing 20 mL of 2% soluble starch and 5 mL buffer was preheated, followed by the addition of 10 mL enzyme solution. Samples were taken at intervals and reacted with iodine solution until the color intensity fell below the standard, and the reaction time (t) was recorded to calculate liquefaction power. (4) Saccharification power was determined using the DNS method. A sample equivalent to 10 g dry basis (±0.01 g) was extracted as described above. Then, 25.0 mL of 2% soluble starch was mixed with 5.0 mL enzyme solution (with NaOH-inactivated control) and incubated at 35 °C for 1 h. The reaction was terminated with NaOH, diluted, and reacted with DNS reagent. After boiling water bath coloration, cooling, and volume adjustment, absorbance was measured at 540 nm. Sugar content was calculated using a glucose standard curve. (5) Fermentation power was determined using a saccharification medium containing 80 g sucrose, 5 g ammonium dihydrogen phosphate, and 5 g potassium dihydrogen phosphate per liter. Aliquots of 100 mL were dispensed into flasks and sterilized at 121 °C. Under sterile conditions, 1 g of Daqu sample (with a blank control) was added, sealed with a fermentation lock containing sulfuric acid, and the initial weight was recorded. Samples were incubated at 30 °C for 72 h, degassed, and reweighed. Fermentation power was calculated based on the weight loss before and after incubation.

### 2.5 DNA extraction and sequencing

During sample preparation, the wheat grains were subjected to direct homogenization under sterile conditions without surface sterilization or dehulling. Subsequently, genomic DNA was extracted from each wheat grain sample using a commercial DNA extraction kit according to the manufacturer’s instructions, and DNA integrity was assessed by agarose gel electrophoresis. Target regions were amplified using specific primers containing unique barcode sequences (~12 bp), the bacterial 16S rRNA gene was amplified using the specific primers 515F (5′- GTGYCAGCMGCCGCGGTAA-3′) and 806R (5′- GGACTACHVGGGTWTCTAAT′). For fungal community analysis, the ITS1 region was amplified using the primers ITS3F (5′- GATGAAGAACGYAGYRAA-3′) and ITS4R (5′- TCCTCCGCTTATTGATATGC-3′), enabling accurate identification of sequences from different samples. PCR amplification was performed in triplicate for each sample, and reactions were terminated during the exponential phase to minimize amplification bias. The resulting amplicons from the same sample were pooled and verified by agarose gel electrophoresis. Target fragments were excised and purified using a gel extraction kit, followed by elution in TE buffer. Purified PCR products were quantified using a Qubit 2.0 fluorometer (Thermo Fisher Scientific). Finally, amplicons from different samples were pooled in equimolar amounts according to sequencing requirements, and sequencing libraries were constructed using an Illumina-compatible library preparation kit.

### 2.6 Sequencing data processing and taxonomic annotation

Raw paired-end sequencing reads were first processed using fastp to remove low-quality reads and adapter sequences. Clean reads were then imported into QIIME2 (Bolyen et al., 2019) using the qiime tools import module for downstream analysis. Primer sequences were removed using the qiime cutadapt trim-paired plugin. Subsequently, paired-end reads were quality filtered and merged using the qiime vsearch merge-pairs function with the following criteria: (i) reads with an average Phred quality score below 20 were discarded; (ii) merged sequences shorter than 50 bp after quality filtering were removed; (iii) up to three mismatches in primer sequences were allowed, and reads containing ambiguous bases were excluded; and (iv) paired reads were merged based on a minimum overlap of 10 bp, and unmerged reads were discarded. Feature sequences were then generated using DADA2. A feature table was constructed and filtered using qiime feature-table filter-features, retaining features with counts greater than 3 and present in more than 2 samples to obtain high-confidence representative sequences. Taxonomic annotation was performed using the qiime feature-classifier module. For 16S rRNA gene sequences, the SILVA database (version 132) clustered at 99% similarity was used. For ITS sequences, the UNITE ITS database (version 10.0) clustered at 99% similarity was applied for fungal taxonomic classification.

### 2.7 Statistical analysis and bioinformatics

Shared and unique microbial taxa among different samples were visualized using an online Venn diagram tool.^1^ Alpha diversity indices, including ACE, Chao1, and Shannon indices, were calculated using the vegan R package (v2.7) to evaluate microbial richness and diversity across sample groups (Oksanen et al., 2026). Beta diversity at the genus level was assessed using unweighted UniFrac distance, and visualized by principal coordinates analysis (PCoA). Random forest analysis (R package randomForest, v4.7) was performed to identify potential microbial biomarkers associated with different wheat-producing regions. Linear discriminant analysis effect size (LEfSe) was conducted via the Galaxy platform^2^ to identify discriminative bacterial taxa across wheat varieties, with an LDA score threshold set at 2 (Segata et al., 2011). Functional prediction of microbial communities was performed using PICRUSt2 based on the Kyoto Encyclopedia of Genes and Genomes (KEGG) database. Bubble plots illustrating associations between key putative functional enzymes and different environments and wheat varieties were generated using the ggplot2 R package (Wickham, 2011). Mantel tests were conducted using the linkET R package (Bellier, 2012) to evaluate correlations between wheat traits, Daqu physicochemical properties, microbial abundance, and KEGG functional profiles. Spearman correlation analysis was performed using the Hmisc R package (Jr et al., 2026) to investigate associations between dominant microbial genera and physicochemical parameters, and interaction networks were visualized using Cytoscape (v3.10.3).

## 3 Results

### 3.1 Sensory quality of Daqu produced from wheat materials cultivated in different regions

**Figure 1:**
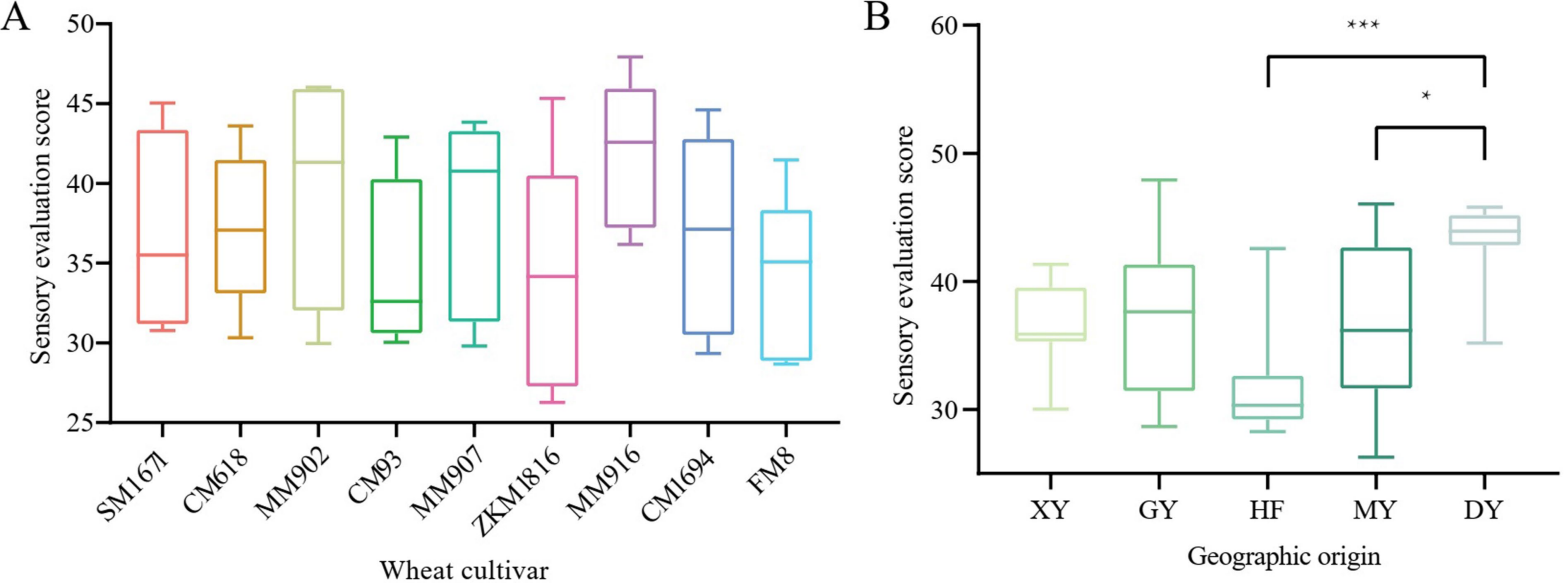
Effects of cultivation environment and wheat cultivar on the sensory quality of Daqu. **(A)** Comparison of sensory evaluation scores of Daqu prepared from different wheat cultivars. Box plots represent the median, interquartile range, and minimum–maximum values of sensory evaluation scores. **(B)** Comparison of sensory evaluation scores of Daqu produced from wheat cultivated in different regions. XY, GY, HF, MY, and DY represent different cultivation environments. Significant differences among regions are indicated by asterisks (*p < 0.05; ***p < 0.001).

A systematic sensory evaluation was conducted on Daqu prepared from nine wheat materials harvested in 2025 to investigate the effects of wheat cultivars and cultivation environment on Daqu sensory quality (Supplementary Table S1). The results showed that, under the same cultivation environment, no significant differences were observed in the sensory quality of Daqu produced from different wheat cultivars (Figure 1A). In contrast, significant differences were detected when the same cultivar was grown in different environments (Figure 1B), indicating that cultivation environment exerted a stronger influence on Daqu sensory characteristics than varietal differences.

Among the tested wheat cultivars, MM916 achieved the highest overall sensory evaluation score (41.77), followed by MM902 (39.45), MM907 (38.00), and CM618 (37.24). Specifically, Daqu produced from MM916 exhibited the highest scores for appearance (8.64), cross-sectional characteristics (19.80), aroma (13.33), and total sensory evaluation (41.77), indicating its superior comprehensive sensory performance. In addition, the brewing-specialized wheat cultivar MM902 also demonstrated favorable sensory characteristics, with relatively high scores in appearance (8.25), cross-sectional characteristics (18.20), aroma (13.00), and overall sensory evaluation (39.45). These results suggest that both MM916 and MM902 possess desirable traits for high-quality Daqu production. Analysis of environmental effects revealed that cultivation environment had a pronounced impact on the sensory quality of Daqu. Wheat harvested from the Dayi cultivation region produced Daqu with significantly higher scores for appearance (8.86), cross-sectional characteristics (20.07), aroma (14.30), and total sensory evaluation (43.23) compared with those from the other four cultivation environments (*p* < 0.05). The superior sensory performance of Daqu derived from wheat grown in Dayi suggests that the environmental conditions in this region are particularly favorable for producing wheat suitable for high-quality Daqu fermentation. These findings further highlight the critical role of environmental factors in determining the brewing quality characteristics of wheat raw materials.

### 3.2 Microbial identification and diversity of different wheat varieties across regions

**Figure 2:**
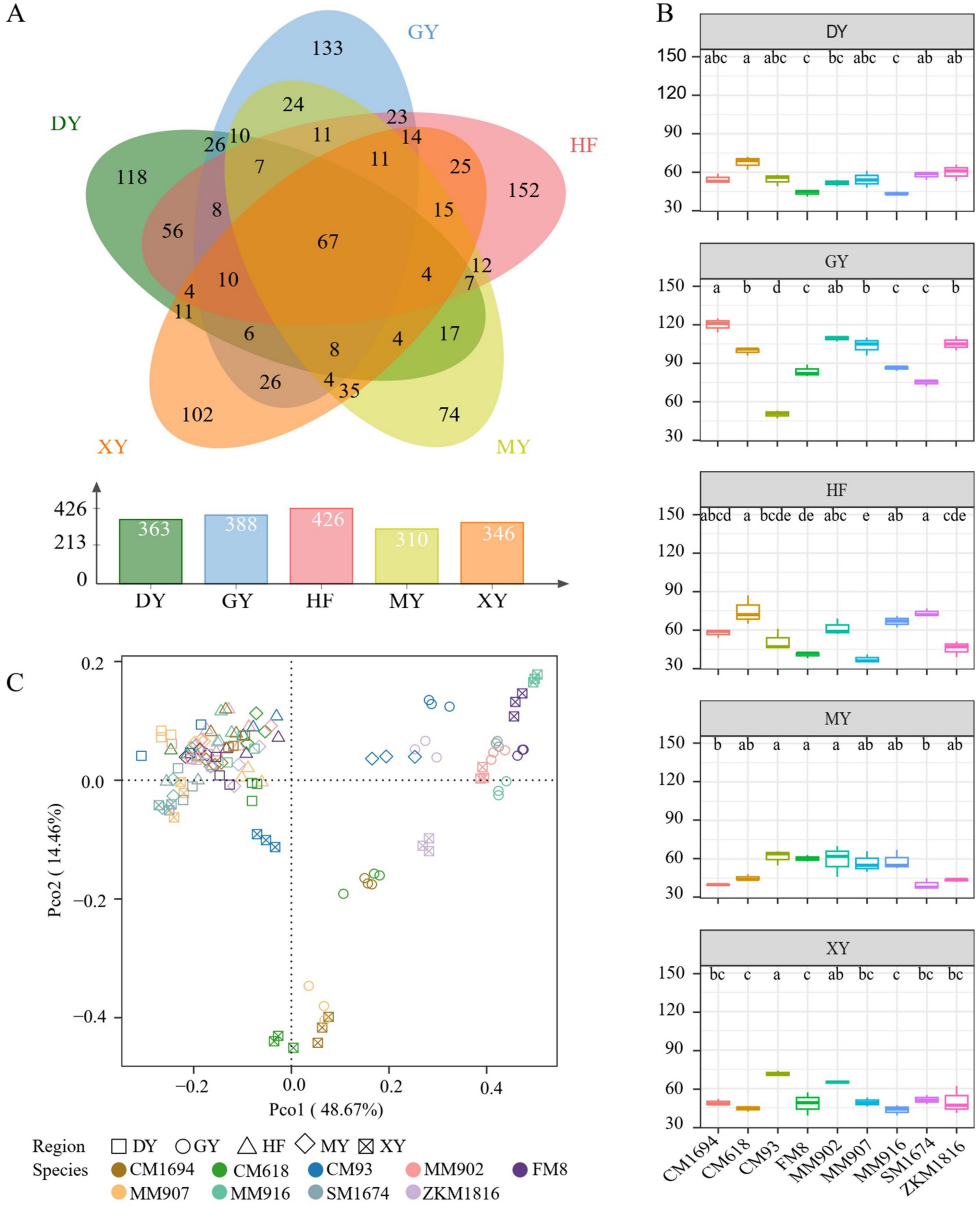
Bacterial identification and diversity of different wheat varieties across regions. **(A)** Venn diagram of bacterial ASV numbers in wheat samples from different regions. **(B)** Alpha diversity of bacterial composition among different wheat varieties across regions. **(C)** Beta diversity of bacterial composition among different wheat varieties across regions.

Characterizing the initial microbial background of wheat raw materials is the logical starting point for elucidating the sources and succession patterns of the biotic drivers in Baijiu starter (Daqu) production. In this study, deep sequencing was performed on 135 wheat samples from 5 regions and 9 varieties, yielding a total of 7.62 Gb of raw data. After quality control, 15,236,279 high-quality sequences were obtained (Supplementary Table S2). A total of 1,732 ASVs were identified in the bacterial community, while only 67 members were shared across the five regions, indicating strong geographic specificity and habitat differentiation (Figure 2A). This pattern was even more pronounced in the fungal community, where only 33 out of 484 ASVs were shared. The extremely low proportion of shared members (4.1 and 6.8%) directly reflects the strong filtering effect of geographic environments on species composition. Among the regions, samples from Hefei exhibited a notable “high diversity” pattern, with the highest total number of bacterial ASVs (430) and unique species (152), as well as relatively high fungal abundance (Supplementary Figure S1), suggesting a more complex microbial niche in this region. As the sample size increased, the species accumulation curves gradually plateaued (Supplementary Figures S2, S3), indicating that the current sequencing depth was sufficient to capture most of the microbial diversity in wheat raw materials. Alpha diversity analysis further revealed that species richness was jointly influenced by region and variety: CM618 showed high diversity adaptability in Dayi and Hefei, while CM1694 and CM93 exhibited dominance in Guiyang and Xinyang, respectively (Figure 2B; Supplementary Table S3). This “variety-region” interaction reflects the selective enrichment of local microbial communities by different wheat varieties. Finally, beta diversity analysis revealed a clear pattern of geographic differentiation. The scatter plot showed distinct clustering of samples by region (Figure 2C), with clear separation among different geographic origins. To further quantify the relative contributions of geographic origin and wheat variety to the raw material microbial community variation, a multi-factor PERMANOVA analysis was performed based on Bray-Curtis distance. The statistical results provided strong quantitative support for our conclusion, revealing that geographic origin was the primary driver of microbial beta diversity, accounting for 37.40% of the total variation (*R*^2^ = 0.3740, *p* = 0.001). In comparison, wheat variety exerted a significant but weaker effect, explaining 13.71% of the community shifts (*R*^2^ = 0.1371, *p* = 0.001). These quantitative lines of evidence robustly support the premise that environmental filtering by geographic origin plays a more dominant role than host selection by variety in shaping the initial microbiota of brewing wheat, notably, Xinyang samples formed a highly independent cluster. These results not only confirm the spatial heterogeneity of community structure but also demonstrate that geographic origin is the primary factor shaping wheat-associated microbial communities.

### 3.3 Taxonomic composition and structure of microbial communities

**Figure 3:**
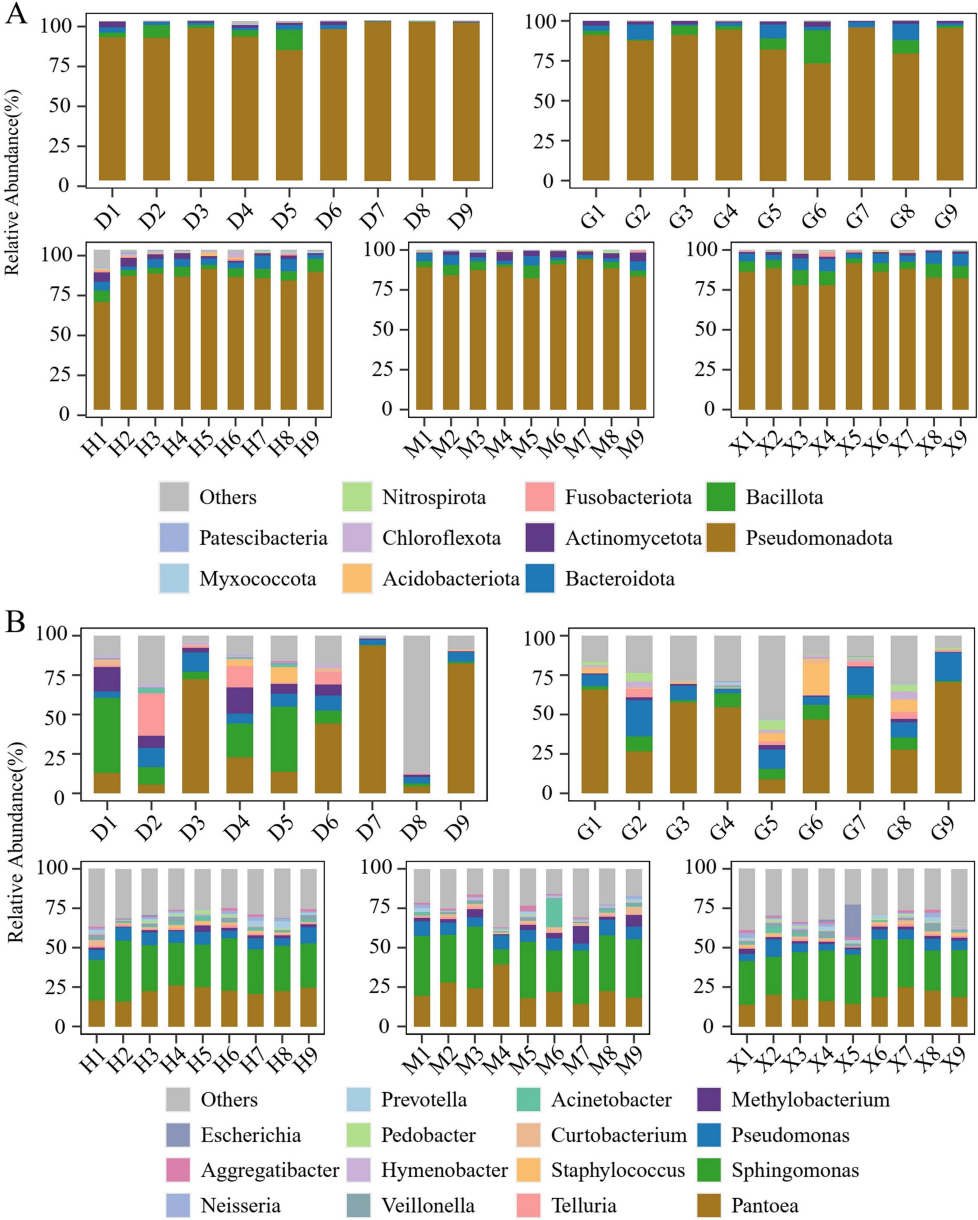
Composition of bacterial community structure. **(A)** Stacked bar plot of the top 10 bacterial taxa at the phylum level. **(B)** Stacked bar plot of the top 15 bacterial taxa at the genus level.

After characterizing the overall patterns of community diversity, analyzing the taxonomic composition of wheat samples from different regions provides a basis for identifying core functional microbial groups. In this study, based on the SILVA and ITS databases, feature sequences were taxonomically annotated at 99% similarity, and the distribution of community structure across different taxonomic levels was evaluated. At the phylum level, the bacterial composition of wheat samples from the five regions showed high similarity. Pseudomonadota dominated across all sampling regions. Within this overall convergent pattern, samples from Guiyang exhibited relatively distinct compositional features: the relative abundances of Bacteroidota, Bacillota, and Actinomycetota were higher than those in the other four regions (Figure 3A), suggesting a selective effect of the local environment in Guiyang on specific bacterial phyla. When the taxonomic resolution shifted from the phylum to the genus level, the previously similar community structures showed clear geographic differentiation. The bacterial communities in Hefei, Mianyang, and Xinyang were relatively similar, with *Pantoea* and *Sphingomonas* as the dominant genus. In contrast, Dayi and Guiyang showed marked divergence at the genus level. Genera such as *Pantoea* and *Methylobacterium* exhibited fluctuations in relative abundance across different varieties and regions (Figure 3B), and their differential distribution represents a major source of heterogeneity in wheat-associated microbial communities. In contrast to the diverse distribution patterns observed in bacterial communities, fungal community composition was relatively consistent across regions. Regardless of geographic origin, fungal taxa were highly enriched in *Ceratobasidium* within the phylum Basidiomycota (Supplementary Figure S4). This pattern, where bacterial communities vary with geography while fungal communities remain relatively stable, suggests that the influence of geographic environment on the initial microbiota of wheat differs among microbial groups. These distribution patterns provide a comprehensive taxonomic overview of wheat-associated microbial communities and reveal the intrinsic relationship between geographic origin and species composition, thereby offering taxonomic clues for the subsequent identification of region-specific biomarkers.

### 3.4 Microbial community characteristics of wheat from different origins

**Figure 4:**
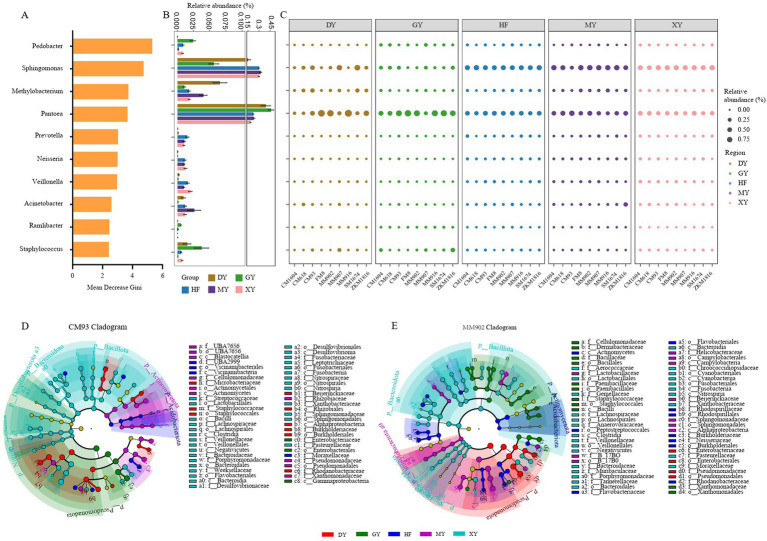
Differences in microbial communities among wheat varieties across regions. **(A)** Importance scores of the top 9 key microbial biomarkers. **(B)** Relative abundances of the top 9 key microbial biomarkers across different environments. **(C)** Bubble plot showing the relative abundances of the top 9 biomarker microorganisms among different wheat varieties across regions. **(D)** Cladogram of differential microbial abundance in CM93 across different regions. **(E)** Cladogram of differential microbial abundance in MM902 across different regions.

Analysis of microbial community composition differences revealed the influence of environmental factors on microbial succession and enabled the identification of functionally relevant microorganisms associated with wheat from different origins. Using a Random Forest model, the relative abundances of bacterial genera across wheat varieties were analyzed, and the top 10 genera (e.g., *Pedobacter*, *Sphingomonas*, *Methylobacterium*, and *Pantoea*) were identified as potential biomarkers distinguishing geographical origins (Figure 4A). These taxa are associated with organic matter degradation and metabolic activity, suggesting roles in early substrate transformation and fermentation initiation. One-way ANOVA showed significant regional differences in these biomarkers. In Guiyang, *Sphingomonas* and *Methylobacterium* were depleted, whereas *Pedobacter*, *Pantoea*, and *Staphylococcus* were enriched (Figure 4B). The enrichment of metabolically versatile genera such as Pseudomonas and *Pantoea*, along with polysaccharide-degrading *Prevotella*, indicates enhanced substrate breakdown and carbon release potential, which may facilitate early fermentation processes. Microbial abundances also varied among wheat varieties within the same region, for example, in Dayi (Sichuan) and Guiyang (Guizhou), the abundance of *Pantoea* in the MM907 variety was lower than that in other varieties (Figure 4C). These results indicate that both geographic environment and wheat variety jointly drive microbial community succession, forming region-specific functional microbial patterns and providing an important basis for understanding microbial dynamics during fermentation.

CM93 and MM902 are the major wheat cultivars used in the production of Sichuan Baijiu. LEfSe analysis (*p* < 0.05, LDA ≥ 3.0) identified 55 and 60 differential taxa across regions for CM93 and MM902, respectively (Supplementary Figures S5, S6). In CM93, *Veillonellaceae* was enriched in Hefei, potentially contributing to organic acid metabolism, while *Actinomycetota* was enriched in Mianyang, suggesting roles in complex substrate degradation and flavor precursor formation (Figure 3D). In MM902, *Bacillota* was enriched in Guiyang, indicating strong enzymatic potential for macromolecule degradation, whereas *Campylobacterota* was enriched in Mianyang, reflecting environmental adaptation. Overall, differences in initial microbial communities among wheat origins may influence both fermentation initiation and downstream metabolic pathways involved in flavor formation.

### 3.5 Functional profiling reveals region- and variety-specific metabolic potential of wheat microbiota

**Figure 5:**
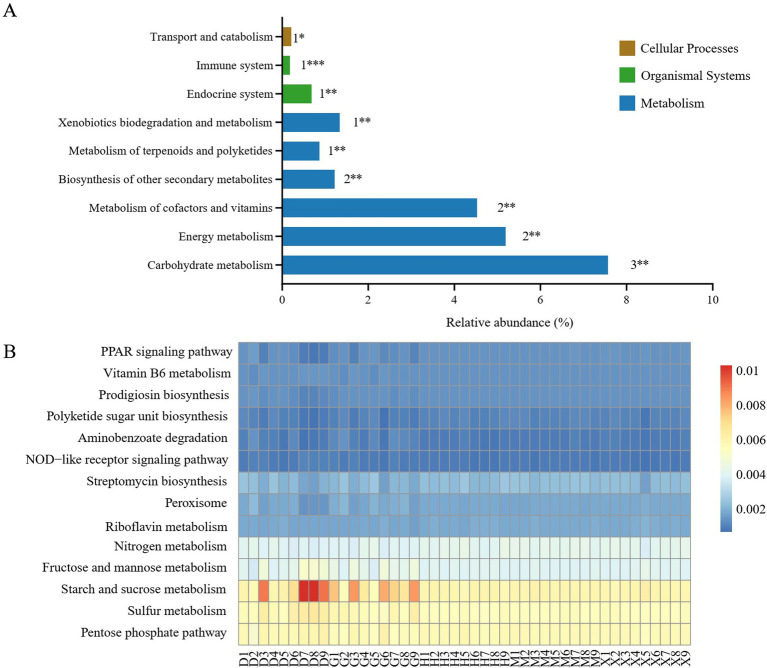
Functional prediction of microbial community composition in wheat samples. **(A)** Overview of predicted functional profiles of microbial communities in wheat raw materials based on PICRUSt2 analysis. Numbers indicate the count of significantly differential KEGG level 3 pathways, and statistical significance is denoted by asterisks (*p < 0.05; **p < 0.01; ***p < 0.001). **(B)** Heatmap showing the relative abundances (>0.1%) of major microbial functional pathways across different wheat varieties and regions, highlighting variation in key metabolic functions.

Building on the observed taxonomic and compositional differences, we further explored the functional potential of wheat-associated microbial communities across regions and varieties. Using PICRUSt2 based on 16S rRNA sequencing data, functional prediction analysis was performed to infer the metabolic capacities of the microbiota. The results showed that a total of 14 significantly differential KEGG level 3 pathways (relative abundance > 0.1%, *p* < 0.05) were identified across 9 wheat varieties from 5 regions (Supplementary Table S4). These pathways were mainly assigned to three level 1 categories: Metabolism, Cellular Processes, and Organismal Systems. Among them, Metabolism was the most dominant functional category, exhibiting the highest relative abundance, indicating highly active microbial metabolic processes in wheat raw materials. Within this category, several level 2 functional groups, including carbohydrate metabolism, energy metabolism, metabolism of cofactors and vitamins, metabolism of terpenoids and polyketides, and xenobiotics biodegradation and metabolism, showed the most significant differences among groups, forming the core functional framework of Daqu-associated microbiota (Figure 5A). At the level 3 pathways, starch and sucrose metabolism, sulfur metabolism, and the pentose phosphate pathway were the most enriched functions. Notably, starch and sucrose metabolism was significantly more abundant in the MM916 and CM1694 varieties from Dayi (Sichuan) compared with other samples (Figure 5B), suggesting that these metabolic pathways play a dominant role in the microbial communities of wheat from specific regions.

### 3.6 Functional prediction of wheat-associated microbial communities related to Daqu fermentation

**Figure 6:**
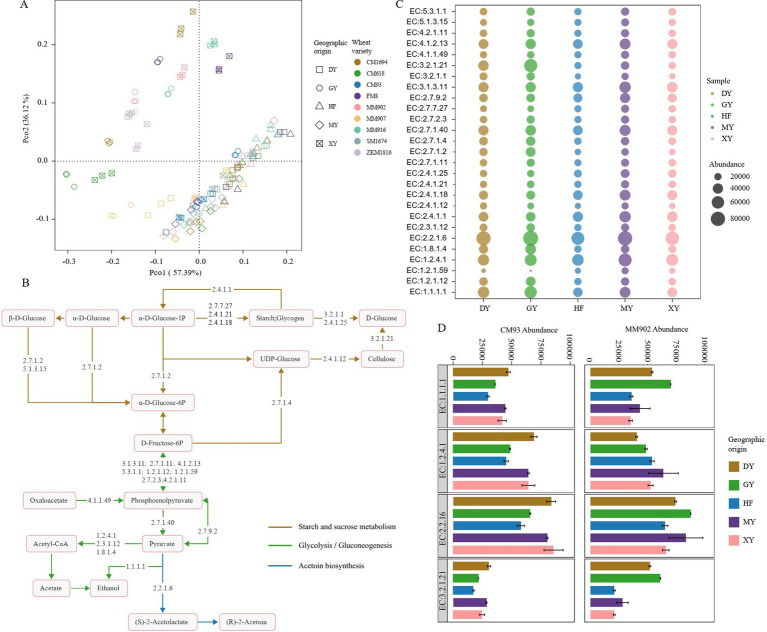
Enzyme profiles of microbial communities in wheat samples across regions and varieties. **(A)** PCoA analysis based on all predicted enzymes, showing overall functional differentiation among samples. **(B)** Profiles of key enzymes associated with starch and sucrose metabolism, glycolysis/gluconeogenesis, and acetoin biosynthesis. **(C)** Relative abundance distribution of enzymes related to starch and sucrose metabolism, glycolysis/gluconeogenesis, and acetoin biosynthesis across different regions. **(D)** Regional variation in the abundance of major enzymes in CM93 and MM902.

Microorganisms naturally associated with wheat grains are considered major sources of Bubble plots illustrating associations between key putative functional enzymes during Daqu fermentation and play critical roles in shaping the metabolic profile and final quality of the fermented product. To investigate the functional potential of wheat-associated microbial communities, PICRUSt2 was employed to predict enzyme-related functional profiles based on microbial community composition. PCoA revealed significant differences in microbial community composition among wheat cultivars and cultivation regions (ANOSIM: *R* = 0.9987, *p* = 0.001; Figure 6A), indicating that both genotype and environmental conditions contributed to the assembly of wheat-associated microbiota. Based on these differences, a total of 27 key enzymes involved in starch and sucrose metabolism, glycolysis, and acetoin biosynthesis pathways were further identified from the KEGG database (Figure 6B; Supplementary Table S5). Functional prediction analysis demonstrated that several core enzymes were enriched across all wheat materials (Figure 6C). These included *β*-glucosidase (EC: 3.2.1.21), which participates in the hydrolysis of *β*-glycosidic bonds within carbohydrate metabolism; acetolactate synthase (EC: 2.2.1.6) and pyruvate dehydrogenase (EC: 1.2.4.1), which are critically associated with acetoin biosynthesis and glycolytic precursor generation, respectively; and alcohol dehydrogenase (EC: 1.1.1.1), a pivotal enzyme driving ethanol metabolism. Alcohol dehydrogenase catalyzes the conversion of pyruvate-derived intermediates into acetaldehyde and ethanol under anaerobic conditions, thereby contributing to flavor compound formation during fermentation, whereas acetolactate synthase (EC: 2.2.1.6) directs the metabolic flux toward acetoin biosynthesis. Notably, the predicted abundances of these enzymes exhibited pronounced spatial heterogeneity across cultivation regions. In particular, alcohol dehydrogenase abundance showed contrasting patterns among regions in the CM93 and MM902 cultivars. Wheat samples of the MM902 cultivar collected from the Guiyang region displayed the highest abundance of alcohol dehydrogenase, whereas the same enzyme exhibited the lowest abundance in CM93 from the same region (Figure 6D). These regional differences in predicted functional microbial potential suggest that cultivation environment may influence the metabolic potential of wheat-associated microbiota, thereby affecting flavor development and sensory characteristics during Daqu fermentation.

### 3.7 Associations Between Wheat Quality Traits and Microbial Community Structure and Function

**Figure 7:**
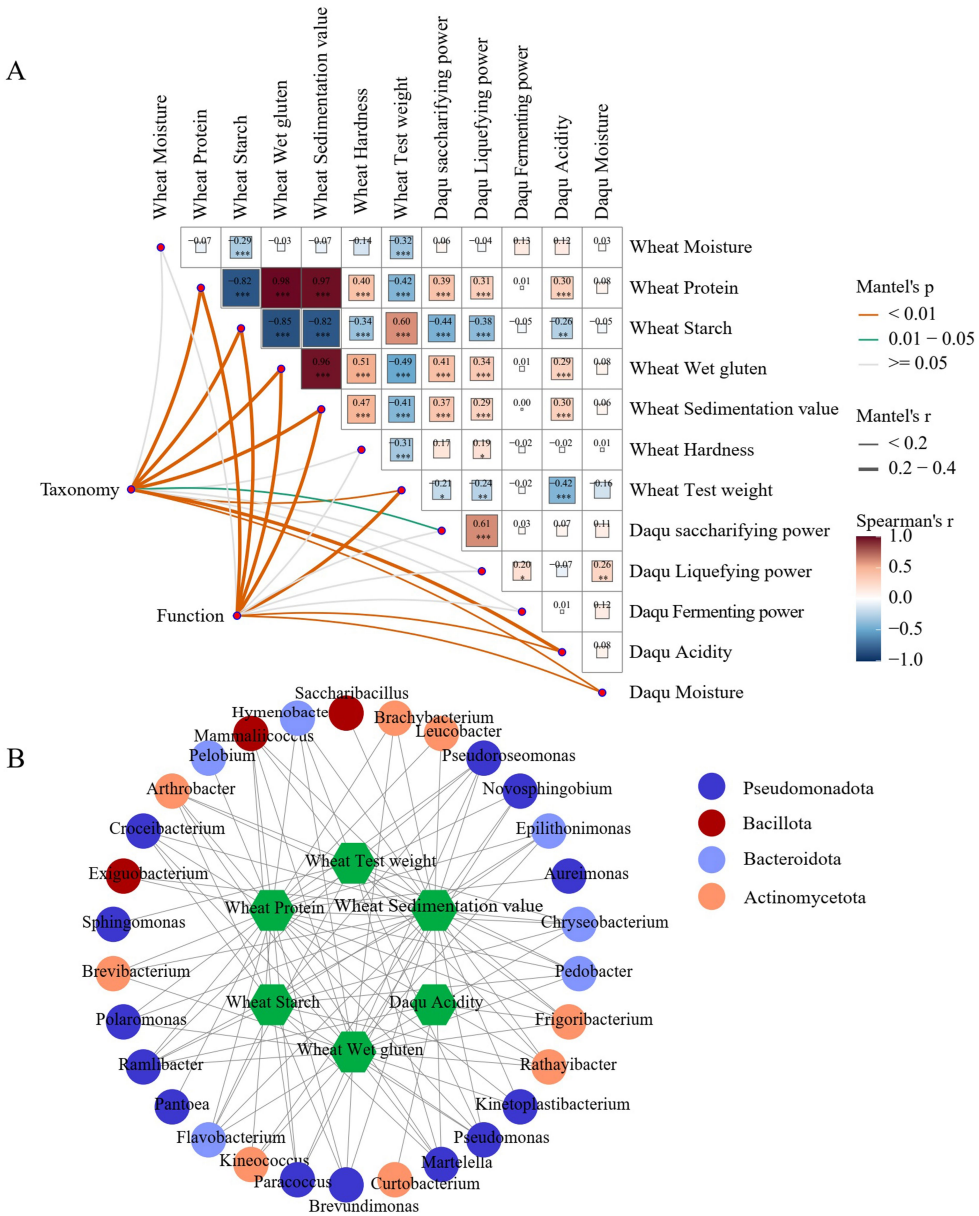
Relationships between wheat-associated microbial communities and physicochemical properties of wheat grains and Daqu. **(A)** Mantel test analysis showing correlations between microbial abundance (taxonomy) and functional profiles with physicochemical properties of wheat grains and Daqu. **(B)** Spearman correlation analysis between microbial community abundance and physicochemical properties of wheat grains.

To further link microbial variation with raw material quality, we examined the associations among wheat grain quality traits, physicochemical properties of the resulting Daqu, microbial community structure, and functional profiles. Using Mantel’s test, significant correlations were identified among these variables (Figure 7A; Supplementary Tables S6, S7). Specifically, both microbial composition and functional abundance were positively correlated with key wheat quality indicators, including protein content (Wheat protein), starch content (Wheat starch), and wet gluten content (Wheat wet gluten; *p* < 0.01; *r* > 0.4), indicating that higher-quality wheat substrates tend to harbor more functionally active and structurally distinct microbial communities. In addition, strong collinearity was observed among wheat physicochemical traits. Protein content, wet gluten content, and sedimentation value were all significantly and positively correlated with each other, whereas starch content showed significant negative correlations with protein, wet gluten, and sedimentation value. This pattern reflects the intrinsic trade-off between storage components in wheat grains and highlights how compositional differences in raw materials may indirectly shape microbial assembly and activity. To further identify key microbial drivers of quality variation, Pearson correlation analysis was conducted between genus-level microbial abundance and wheat quality traits (Figure 7B). A total of 32 genera, including *Sphingomonas*, *Pedobacter*, and *Martelella*, showed significant correlations with wheat quality indicators. These taxa mainly belonged to Pseudomonadota, Bacillota, Bacteroidota, and Actinomycetota, suggesting that multiple phylogenetic groups contribute to quality-associated functional processes. Collectively, these results indicate that core microbial taxa are closely linked to wheat grain quality and may act as key biological regulators affecting the formation and physicochemical properties of Daqu. This finding not only reveals the intrinsic connection between raw material quality and microbial ecology, but also suggests that specific microbial signatures could serve as potential indicators for evaluating and selecting high-quality brewing wheat, thereby providing a new perspective for optimizing raw material selection and improving fermentation outcomes.

## 4 Discussion

Wheat grain quality is significantly driven by the interaction between genotype and cultivation environment (Martínez-Peña et al., 2023), and these physicochemical properties further determine its suitability in food processing and the quality performance of derived products (Kaplan Evlice, 2022). In traditional Baijiu Daqu production, wheat raw materials are typically used without sterilization, allowing the indigenous microorganisms they carry to rapidly colonize during the early stages of starter fermentation and gradually gain ecological dominance in the complex open fermentation environment, thereby profoundly shaping the assembly and succession of Daqu microbial communities (Jin et al., 2017; Du et al., 2019). Although previous studies have elucidated the impact of wheat physicochemical properties on Daqu quality, the synergistic effects of wheat variety and cultivation environment in shaping the microbial community structure and potential functions of raw materials remain insufficiently understood. In this study, high-throughput sequencing of 16S rRNA and ITS regions was applied to systematically analyze 135 wheat samples from five representative regions and nine wheat varieties, identifying 1,732 bacterial ASVs and 484 fungal ASVs. Alpha and beta diversity analyses revealed significant differences in microbial community structures among regions and varieties, indicating strong environmental differentiation and host selection effects. These findings are consistent with those of Han et al., who reported that environmental factors and raw material composition are key drivers of Daqu microbial community assembly (Han et al., 2024). Notably, samples from Hefei exhibited higher ASV richness and a greater proportion of unique taxa, suggesting that local conditions such as humid climate, moderate temperature, and differences in agricultural management practices may provide more complex ecological niches that facilitate microbial diversification. This “high diversity-high specificity” pattern implies that wheat from different origins already carries distinct initial microbial reservoirs prior to entering the fermentation system, which may subsequently influence Daqu microbiome assembly through a “grain-to-microbiota transmission” mechanism. Furthermore, such geographically and varietally driven differences in initial microbial communities not only determine the foundational microbial composition but may also influence substrate utilization capacity, activation of metabolic pathways, and microbial interaction networks, thereby affecting the functional stability and flavor-forming potential of the Daqu fermentation system. Therefore, wheat raw materials should not be regarded merely as sources of carbon and nitrogen, but rather as carriers of functional microbial communities that play an integral role in shaping the ecological assembly of Daqu.

The microbial composition of wheat raw materials is influenced by multiple factors. Through taxonomic annotation, this study found that although microbial diversity exhibited clear geographic variation, a conserved core microbiota was consistently identified across all samples, dominated by Pseudomonadota and Basidiomycota. These groups are also commonly enriched in wheat rhizosphere communities, where they contribute to nitrogen cycling and organic matter decomposition, thereby indirectly influencing wheat quality (Zhang et al., 2026). At the genus level, *Pantoea*, *Methylobacterium*, and *Pseudomonas* were widely distributed across multiple regions, indicating their ecological adaptability and stability in wheat-associated environments. Previous studies have also reported genera such as *Staphylococcus*, *Pantoea*, *Alternaria*, and *Mycosphaerella* as dominant endophytic taxa in wheat (Zhang et al., 2022). The overlap and divergence among these dominant genera suggest the presence of a broadly distributed core microbiota, which likely constitutes the initial microbial seed bank for Daqu fermentation. The coexistence of this universal microbial background with region-specific abundance patterns may jointly determine the early-stage microbial trajectories during starter fermentation. In addition, the biomarkers identified by the Random Forest model provide important ecological insights. For instance, *Pantoea* and *Pseudomonas* have been reported to produce organic acids such as acetic acid, citric acid, gluconic acid, succinic acid, and malic acid, which can influence wheat quality and potentially modulate the early fermentation environment (Zhang et al., 2022). More importantly, distinct taxa identified in the same wheat varieties across different regions, such as Bacillota and Actinomycetota in CM93 and MM902, highlight the strong effect of environmental filtering. Members of Bacillota are capable of forming endospores, enabling survival under stress conditions such as desiccation and nutrient limitation, and conferring enhanced heat resistance. In contrast, Actinomycetota are known for their ability to solubilize phosphorus in both acidic and alkaline soils, thereby improving nutrient availability; wheat growth has been shown to be more favorable in soils with pH ranging from 6.0 to 9.0 compared to extreme conditions (Han et al., 2024). Furthermore, metabolites produced by Actinomycetota contribute to key aroma and flavor compounds in Baijiu (Chen et al., 2022; Feng et al., 2023). These findings demonstrate that even within the same wheat variety, shifts in geographic environmental niches can reshape the initial microbial composition of raw materials, which in turn may influence the microbial assembly and functional potential of Daqu, ultimately affecting its quality.

Differences in microbial community structure are ultimately reflected in shifts in functional metabolic potential. Based on PICRUSt2 prediction, although wheat varieties from different geographic origins showed compositional variation, their microbiota consistently exhibited highly active metabolic profiles, with “Metabolism” as the dominant level 1 functional category. At the level 2 pathways, carbohydrate metabolism, energy metabolism, and metabolism of cofactors and vitamins were significantly enriched. Among these, carbohydrate metabolism represents the core functional module in Daqu fermentation (Ping et al., 2020) and serves as a major source of flavor precursors, including alcohols, esters, phenols, and organic acids (Chen et al., 2021; Ma et al., 2022). Traditional perspectives often attribute the production of putative functional enzymes in Daqu primarily to microbial proliferation and secretion during fermentation (Han et al., 2023; Xia et al., 2023). However, our results suggest that wheat-associated microbiota already harbor a substantial reservoir of key metabolic enzymes prior to fermentation. These include enzyme systems involved in starch and sucrose metabolism, glycolysis, and acetoin biosynthesis, indicating that raw material microbiota possess intrinsic biochemical activity before entering the fermentation system. Furthermore, quantitative prediction of 27 key enzymes revealed clear region- and variety-associated patterns. For example, *β*-glucosidase (EC: 3.2.1.21) and alcohol dehydrogenase (EC: 1.1.1.1) showed significant geographic specificity in CM93 and MM902, whereas acetolactate synthase (EC: 2.2.1.6) exhibited strong variety preference, being significantly enriched only across regions within CM93. These enzymes are involved in the transformation of organic acids such as lactic acid, acetic acid, and propionic acid, thereby influencing ethanol production efficiency. Notably, acetoin, a key intermediate in these pathways, serves as an important precursor for tetramethylpyrazine (ligustrazine), which contributes to nutty and roasted aroma characteristics and enhances flavor complexity and balance in Baijiu (Lourencetti et al., 2018; Xu et al., 2018; Rutkis et al., 2021; Wang et al., 2025). These findings indicate that variation in enzyme abundance pre-configured within wheat-associated microbiota provides distinct biochemical backgrounds for different regions and varieties. Such differences may influence the accumulation of early-stage metabolites during fermentation, thereby shaping the trajectory of metabolic processes and ultimately contributing to the regional characteristics of Daqu flavor and quality.

Wheat grains not only serve as the physical substrate for Daqu fermentation, but their physicochemical composition also shapes a distinct nutrient landscape that exerts selective pressure on the initial microbial community. In this study, Mantel test analysis demonstrated that key grain quality traits, particularly protein, starch, and wet gluten content, are major environmental drivers shaping both microbial community structure and functional potential. Pearson correlation analysis further identified 22 key genera significantly associated with wheat quality indicators, among which *Sphingomonas* and *Martelella* showed strong positive correlations with protein and wet gluten content. As widely distributed taxa in plant-associated and rhizosphere environments, *Sphingomonas* has been reported to promote seed germination, maintain stomatal function, and enhance chlorophyll content, thereby improving plant physiological performance (Wang et al., 2022). The strong association observed here suggests that such functional genera may be closely linked to the accumulation of protein-related substrates during wheat development, while also acting as pre-enriched bioactive components in raw materials. Upon entering the fermentation system, these microorganisms may leverage their metabolic capabilities to facilitate the conversion of nutrient substrates into key flavor precursors during the early stages of Daqu production. This interconnected relationship among wheat quality, microbial composition, and functional potential highlights a potential ecological linkage where grain quality is associated with variations in Daqu fermentation outcomes through its correlation with the initial microbiota. These findings provide a mechanistic basis for understanding how raw material traits are mirrored in fermentation performance and suggest that specific microbial signatures associated with grain quality could serve as potential indicators for the selection of high-quality brewing wheat.

However, several limitations in the current study should be acknowledged, which also point to important directions for future research. Although geographic origin was identified as a primary driver shaping the microbial community and functional preconfiguration of wheat raw materials, the specific underlying environmental variables require deeper investigation. In this study, regional factors were primarily treated as broad ecological backgrounds. In reality, soil physicochemical properties (such as pH, organic matter content, and available nitrogen/phosphorus/potassium) and real-time meteorological conditions (including precise diurnal temperature variations, cumulative precipitation, and relative humidity during the grain-filling stage) are critical forces driving plant-microbe interactions and vertical transmission. Future investigations incorporating detailed soil properties and high-resolution meteorological data across different Daqu wheat-producing regions will be essential to decouple the specific environmental filters influencing raw material functionality. Moreover, tracking the dynamic succession from these initial wheat-associated microbial reservoirs to the actual Daqu fermentation process will provide direct mechanistic insights into the ‘grain-to-microbiota’ transmission and its ultimate impact on Baijiu quality.

In summary, this study systematically characterized the microbial background of wheat raw materials from five major producing regions in China, demonstrating that wheat serves not only as a physical substrate for Daqu fermentation but also as a direct source of core functional microorganisms and key enzyme systems. Our results show that geographic origin and host variety jointly shape the microbial community through environmental filtering, with Hefei samples exhibiting the highest community diversity, while pronounced beta diversity highlights strong heterogeneity in initial microbial assemblages. These community differences further lead to spatial variation in key metabolic pathways, particularly carbohydrate metabolism and acetoin biosynthesis, as well as in associated enzymes such as alcohol dehydrogenase and *β*-glucosidase. This forms a “functionally preconfigured” microbial system in raw materials, which may predetermine the trajectory of flavor metabolism prior to fermentation onset. In addition, wheat quality traits (especially protein, starch, and wet gluten content) create specific nutrient landscapes that selectively enrich functional genera such as *Sphingomonas* and *Martelella*. This integrated relationship among grain quality, microbial community, and functional potential provides a biological perspective on the essence of high-quality brewing materials, highlighting that wheat acts not only as an energy source but also as an initial driver of Daqu fermentation. These findings offer a novel microbiome-based dimension for the geographical traceability and quality evaluation of brewing wheat, and establish a theoretical foundation for the precise control of Baijiu Daqu fermentation.

## Data Availability

The datasets generated and/or analyzed during this study are available in the National Center for Biotechnology Information BioProject repository under accession number PRJNA1465074 (https://www.ncbi.nlm.nih.gov/bioproject/PRJNA1465074).

## Footnotes

1
https://jvenn.toulouse.inra.fr/app/example.html

2
http://galaxy.biobakery.org/
