# Supplementary material for: Geographic origin and wheat variety shape microbial and functional profiles of brewing wheat for Daqu fermentation
Source: Front Microbiol. 2026 Jul 24;17:1879363. doi: 10.3389/fmicb.2026.1879363 (PMC13447316; doi:10.3389/fmicb.2026.1879363)
Supplement: Supplementary file 1 [file Image_1.pdf]

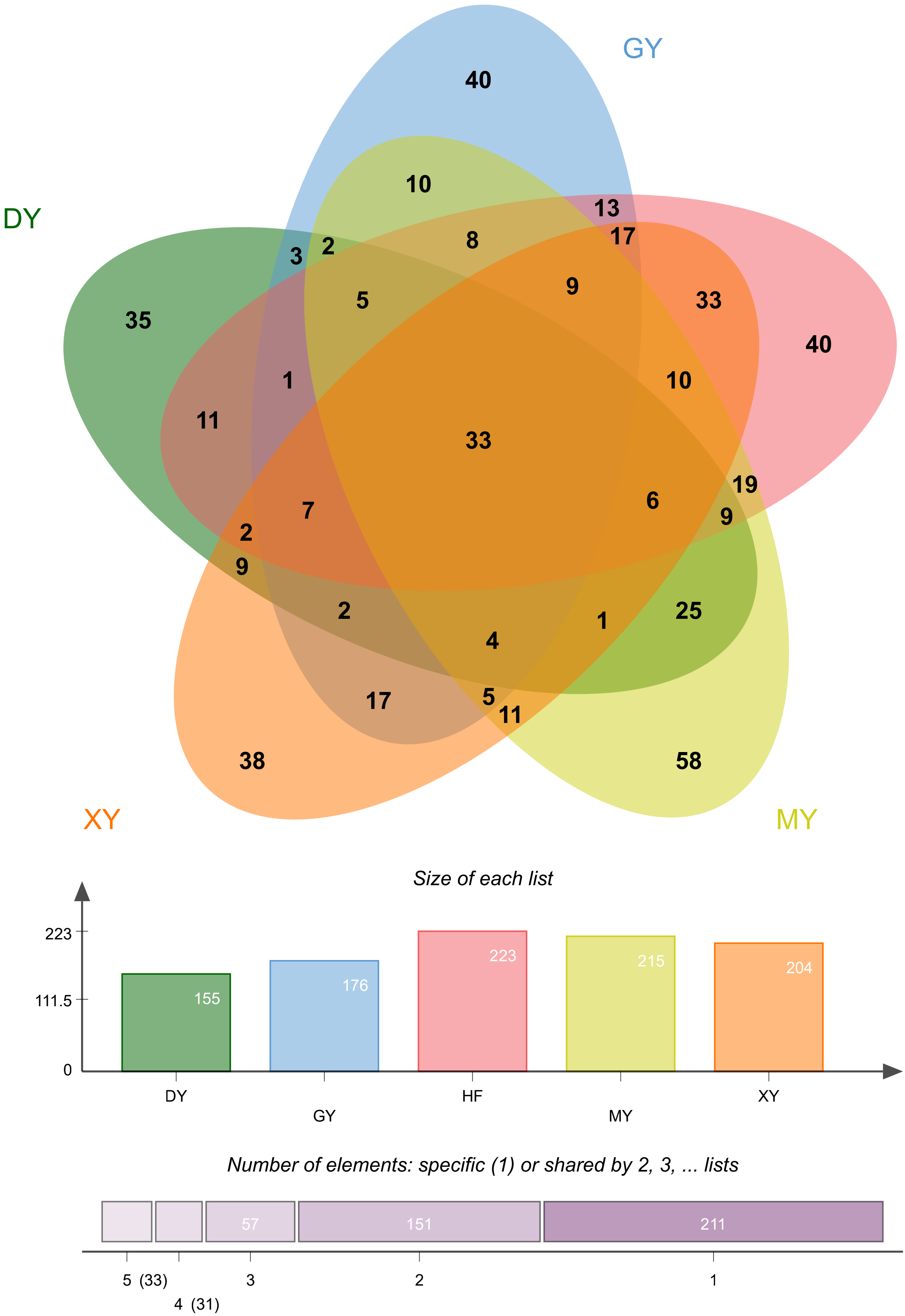

GY
DY
HF
XY
MY
40
10
13
17
2
3
8
35
5
9
33
40
1
10
11
33
7
6
19
2
9
9
2
1
25
4
5
11
17
38
58
Size of each list
223
111.5
0
155
176
223
215
204
DY
GY
HF
MY
XY
Number of elements: specific (1) or shared by 2, 3, ... lists
57
151
211
5 (33)
4 (31)
3
2
1
