# Supplementary material for: Geographic origin and wheat variety shape microbial and functional profiles of brewing wheat for Daqu fermentation
Source: Front Microbiol. 2026 Jul 24;17:1879363. doi: 10.3389/fmicb.2026.1879363 (PMC13447316; doi:10.3389/fmicb.2026.1879363)
Supplement: Supplementary file 2 [file Image_2.pdf]

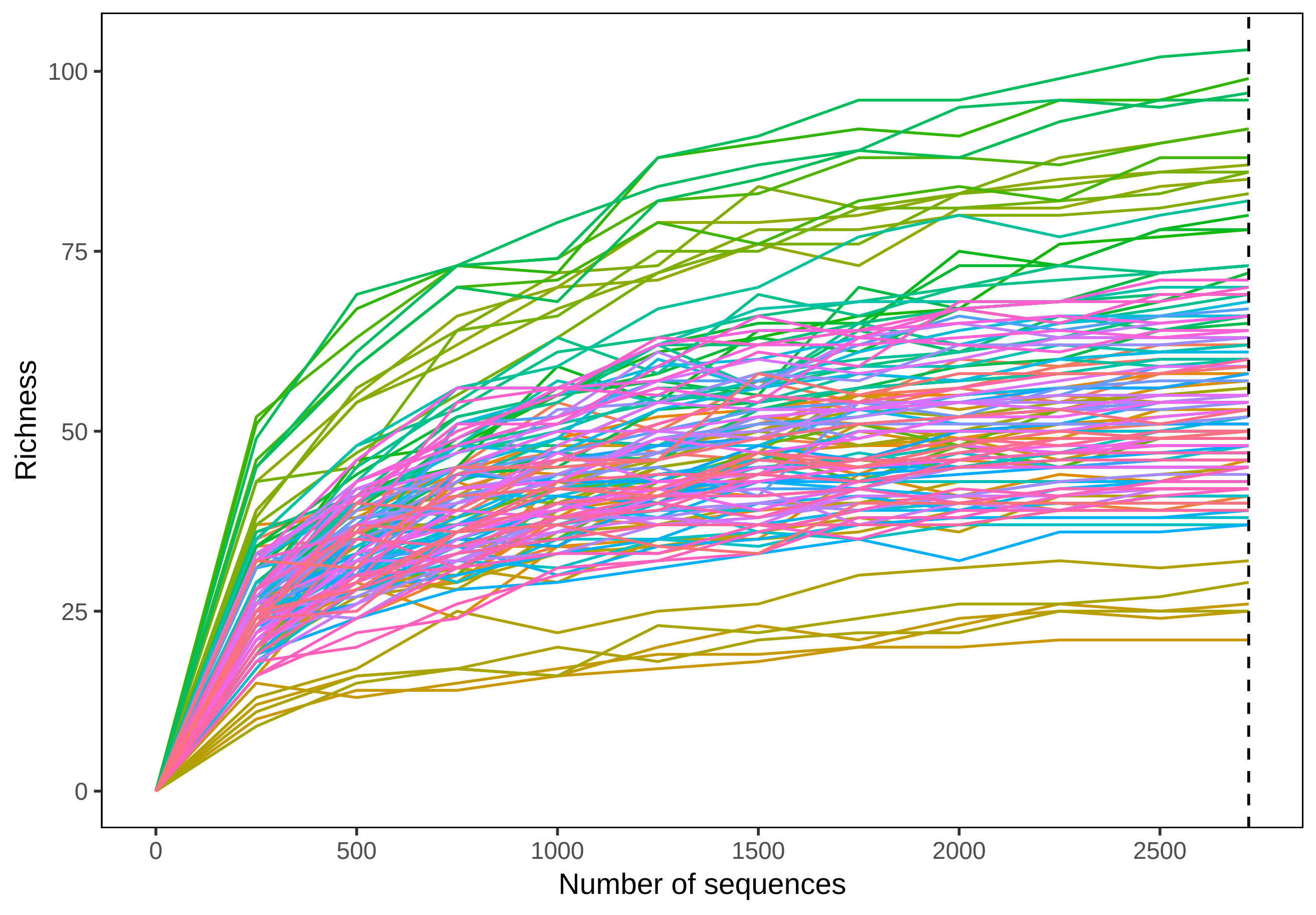

Richness
100
75
50
25
0
0
500
1000
1500
2000
2500
Number of sequences
