# Supplementary material for: Geographic origin and wheat variety shape microbial and functional profiles of brewing wheat for Daqu fermentation
Source: Front Microbiol. 2026 Jul 24;17:1879363. doi: 10.3389/fmicb.2026.1879363 (PMC13447316; doi:10.3389/fmicb.2026.1879363)
Supplement: Supplementary file 3 [file Image_3.pdf]

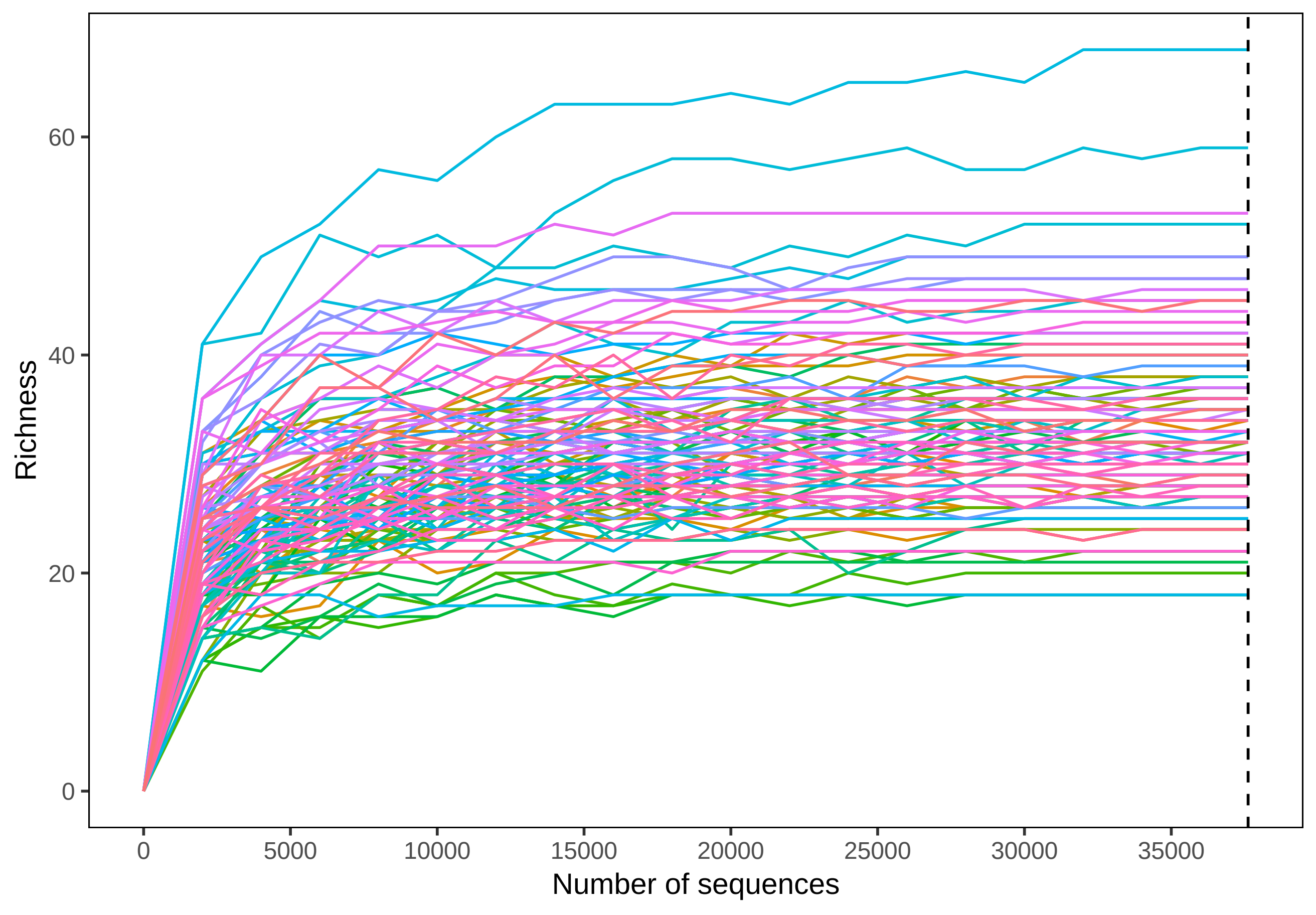
Richness
60
40
20
0
0
5000
10000
15000
20000
25000
30000
35000
Number of sequences
