# Supplementary material for: Geographic origin and wheat variety shape microbial and functional profiles of brewing wheat for Daqu fermentation
Source: Front Microbiol. 2026 Jul 24;17:1879363. doi: 10.3389/fmicb.2026.1879363 (PMC13447316; doi:10.3389/fmicb.2026.1879363)
Supplement: Supplementary file 4 [file Image_4.pdf]

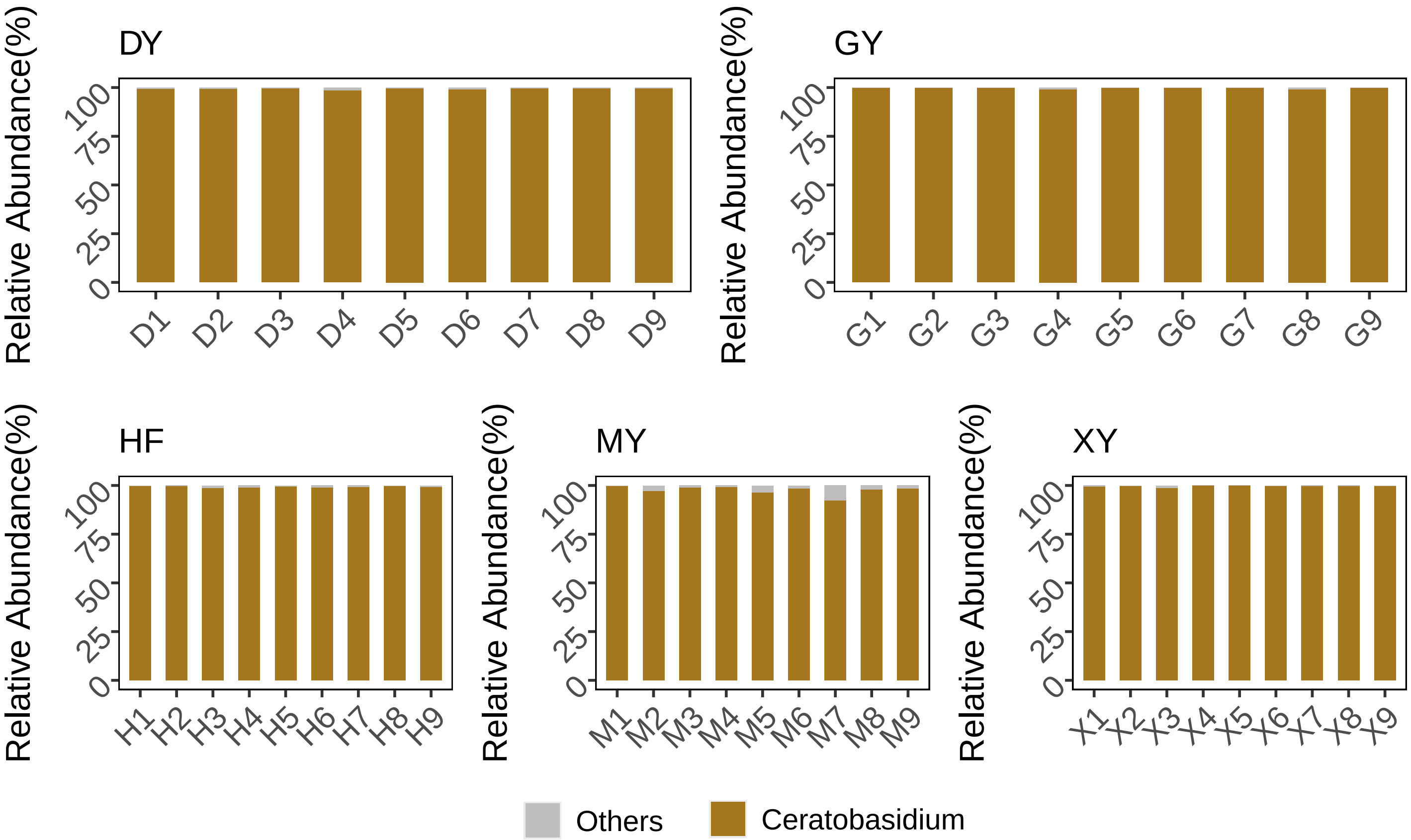

DY
GY
HF
MY
XY
Relative Abundance(%)
0
25
50
75
100
D1
D2
D3
D4
D5
D6
D7
D8
D9
G1
G2
G3
G4
G5
G6
G7
G8
G9
H1
H2
H3
H4
H5
H6
H7
H8
H9
M1
M2
M3
M4
M5
M6
M7
M8
M9
X1
X2
X3
X4
X5
X6
X7
X8
X9
Others
Ceratobasidium
