# Supplementary material for: Geographic origin and wheat variety shape microbial and functional profiles of brewing wheat for Daqu fermentation
Source: Front Microbiol. 2026 Jul 24;17:1879363. doi: 10.3389/fmicb.2026.1879363 (PMC13447316; doi:10.3389/fmicb.2026.1879363)
Supplement: Supplementary file 5 [file Image_5.pdf]

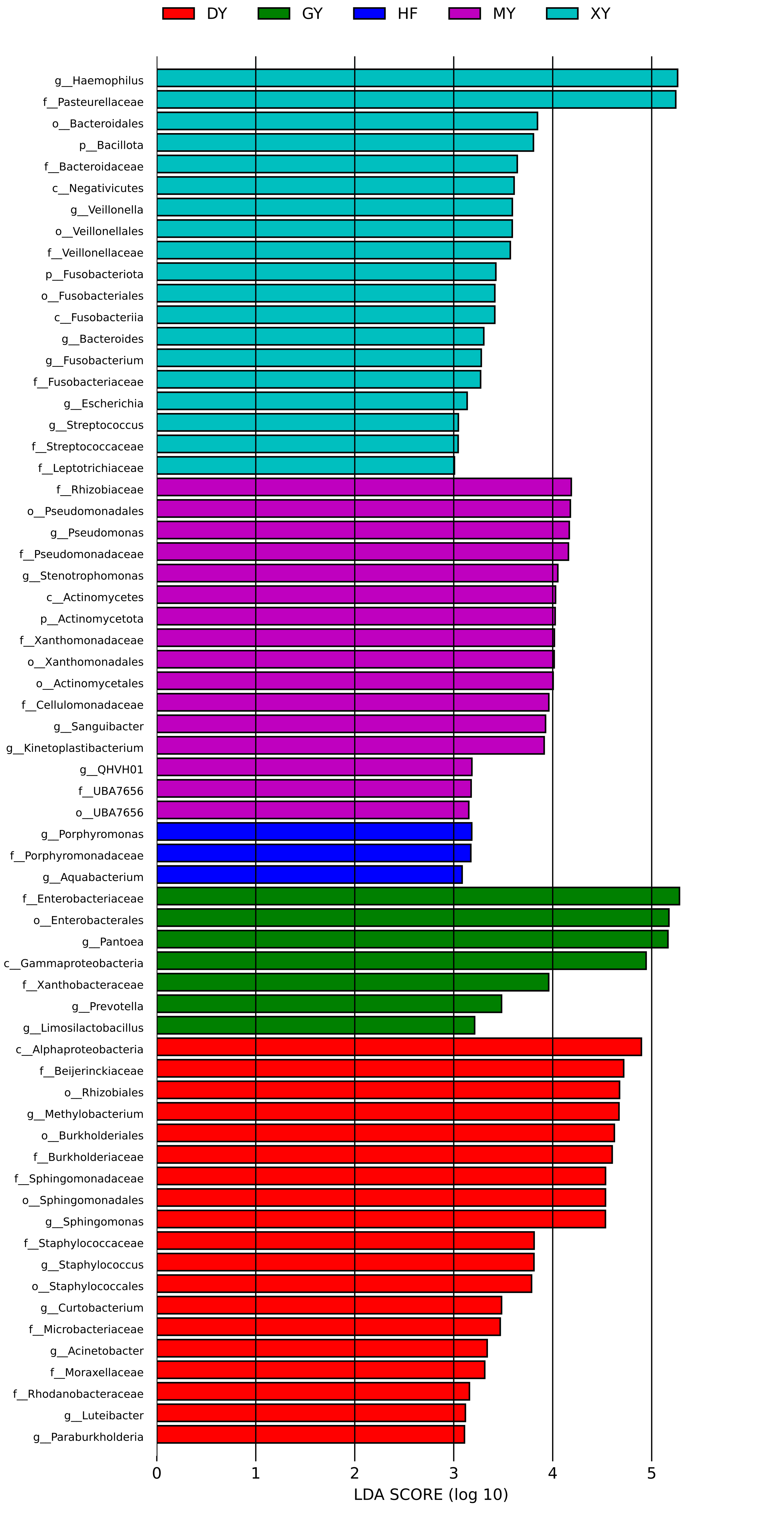

DY
GY
HF
MY
XY
g__Haemophilus
f__Pasteurellaceae
o__Bacteroidales
p__Bacillota
f__Bacteroidaceae
c__Negativicutes
g__Veillonella
o__Veillonellales
f__Veillonellaceae
p__Fusobacteriota
o__Fusobacteriales
c__Fusobacteriia
g__Bacteroides
g__Fusobacterium
f__Fusobacteriaceae
g__Escherichia
g__Streptococcus
f__Streptococcaceae
f__Leptotrichiaceae
f__Rhizobiaceae
o__Pseudomonadales
g__Pseudomonas
f__Pseudomonadaceae
g__Stenotrophomonas
c__Actinomycetes
p__Actinomycetota
f__Xanthomonadaceae
o__Xanthomonadales
o__Actinomycetales
f__Cellulomonadaceae
g__Sanguibacter
g__Kinetoplastibacterium
g__QHVH01
f__UBA7656
o__UBA7656
g__Porphyromonas
f__Porphyromonadaceae
g__Aquabacterium
f__Enterobacteriaceae
o__Enterobacterales
g__Pantoea
c__Gammaproteobacteria
f__Xanthobacteraceae
g__Prevotella
g__Limosilactobacillus
c__Alphaproteobacteria
f__Beijerinckiaceae
o__Rhizobiales
g__Methylobacterium
o__Burkholderiales
f__Burkholderiaceae
f__Sphingomonadaceae
o__Sphingomonadales
g__Sphingomonas
f__Staphylococcaceae
g__Staphylococcus
o__Staphylococcales
g__Curtobacterium
f__Microbacteriaceae
g__Acinetobacter
f__Moraxellaceae
f__Rhodanobacteraceae
g__Luteibacter
g__Paraburkholderia
0
1
2
3
4
5
LDA SCORE (log 10)
