# Supplementary material for: Geographic origin and wheat variety shape microbial and functional profiles of brewing wheat for Daqu fermentation
Source: Front Microbiol. 2026 Jul 24;17:1879363. doi: 10.3389/fmicb.2026.1879363 (PMC13447316; doi:10.3389/fmicb.2026.1879363)
Supplement: Supplementary file 6 [file Image_6.pdf]

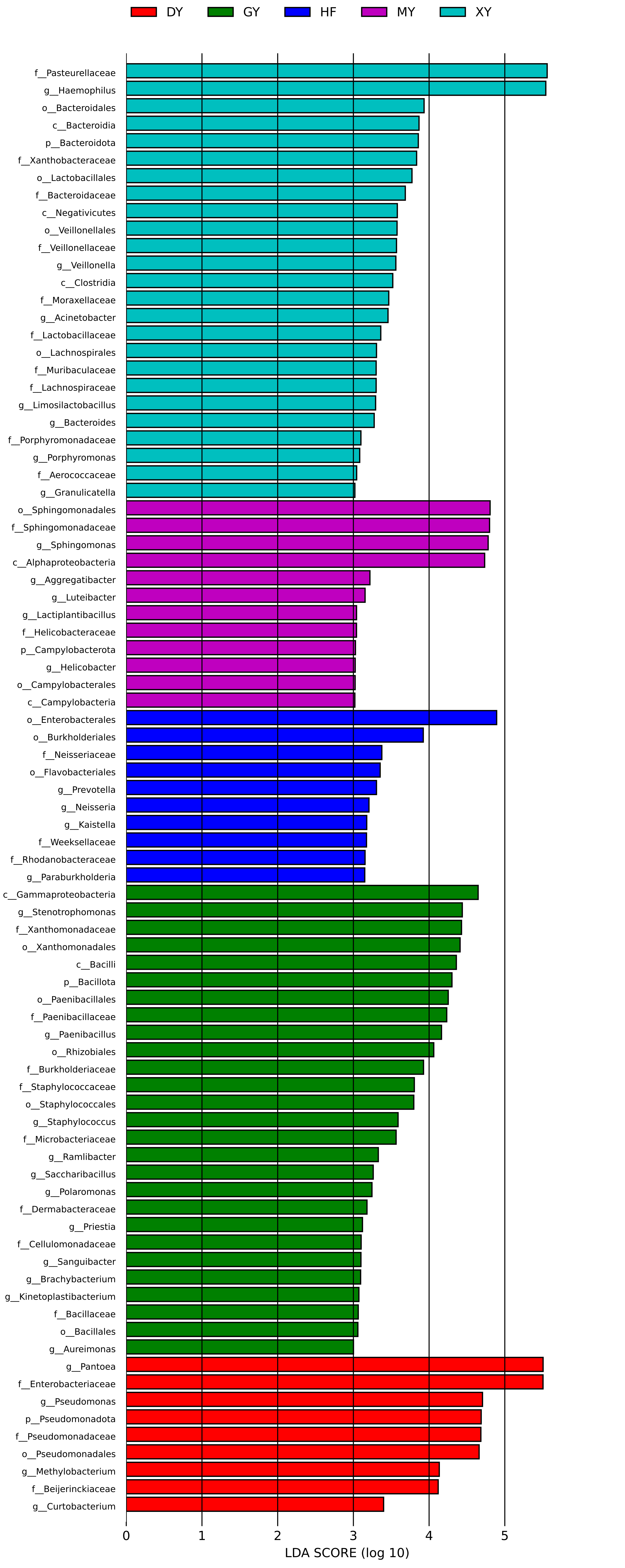

DY
GY
HF
MY
XY
f__Pasteurellaceae
g__Haemophilus
o__Bacteroidales
c__Bacteroidia
p__Bacteroidota
f__Xanthobacteraceae
o__Lactobacillales
f__Bacteroidaceae
c__Negativicutes
o__Veillonellales
f__Veillonellaceae
g__Veillonella
c__Clostridia
f__Moraxellaceae
g__Acinetobacter
f__Lactobacillaceae
o__Lachnospirales
f__Muribaculaceae
f__Lachnospiraceae
g__Limosilactobacillus
g__Bacteroides
f__Porphyromonadaceae
g__Porphyromonas
f__Aerococcaceae
g__Granulicatella
o__Sphingomonadales
f__Sphingomonadaceae
g__Sphingomonas
c__Alphaproteobacteria
g__Aggregatibacter
g__Luteibacter
g__Lactiplantibacillus
f__Helicobacteraceae
p__Campylobacterota
g__Helicobacter
o__Campylobacterales
c__Campylobacteria
o__Enterobacterales
o__Burkholderiales
f__Neisseriaceae
o__Flavobacteriales
g__Prevotella
g__Neisseria
g__Kaistella
f__Weeksellaceae
f__Rhodanobacteraceae
g__Paraburkholderia
c__Gammaproteobacteria
g__Stenotrophomonas
f__Xanthomonadaceae
o__Xanthomonadales
c__Bacilli
p__Bacillota
o__Paenibacillales
f__Paenibacillaceae
g__Paenibacillus
o__Rhizobiales
f__Burkholderiaceae
f__Staphylococcaceae
o__Staphylococcales
g__Staphylococcus
f__Microbacteriaceae
g__Ramlibacter
g__Saccharibacillus
g__Polaromonas
f__Dermabacteraceae
g__Priestia
f__Cellulomonadaceae
g__Sanguibacter
g__Brachybacterium
g__Kinetoplastibacterium
f__Bacillaceae
o__Bacillales
g__Aureimonas
g__Pantoea
f__Enterobacteriaceae
g__Pseudomonas
p__Pseudomonadota
f__Pseudomonadaceae
o__Pseudomonadales
g__Methylobacterium
f__Beijerinckiaceae
g__Curtobacterium
0
1
2
3
4
5
LDA SCORE (log 10)
